# Automated detection of spreading depolarizations in electrocorticography

**DOI:** 10.1038/s41598-025-91623-7

**Published:** 2025-03-12

**Authors:** Sreekar Puchala, Ethan Muchnik, Anca Ralescu, Jed A. Hartings

**Affiliations:** 1 Department of Computer Science, University of Cincinnati, Cincinnati, OH 45267 USA; 2 Department of Electrochemistry, University of Oregon, Eugene, OR USA; 3 Department of Neurosurgery, University of Cincinnati College of Medicine, 231 Albert Sabin Way, Cincinnati, OH 45267 USA

**Keywords:** Spreading depolarization, Spreading depression, Electroencephalography, Machine learning, Automated detection, Computational algorithm, Neurocritical care, Brain injuries, Translational research, Neuronal physiology

## Abstract

Spreading depolarizations (SD) in the cerebral cortex are a novel mechanism of lesion development and worse outcomes after acute brain injury, but accurate diagnosis by neurophysiology is a barrier to more widespread application in neurocritical care. Here we developed an automated method for SD detection by training machine-learning models on electrocorticography data from a 14-patient cohort that included 1,548 examples of SD direct-current waveforms as identified in expert manual scoring. As determined by leave-one-patient-out cross-validation, optimal performance was achieved with a gradient-boosting model using 30 features computed from 400-s electrocorticography segments sampled at 0.1 Hz. This model was applied to continuous electrocorticography data by generating a time series of SD probability [*P*_*SD*_*(t)*], and threshold *P*_*SD*_*(t)* values to trigger SD predictions were determined empirically. The developed algorithm was then tested on a novel dataset of 10 patients, resulting in 1,252 true positive detections (/1,953; 64% sensitivity) and 323 false positives (6.5/day). Secondary manual review of false positives showed that a majority (224, or 69%) were likely real SDs, highlighting the conservative nature of expert scoring and the utility of automation. SD detection using sparse sampling (0.1 Hz) is optimal for streaming and use in cloud computing applications for neurocritical care.

## Introduction

Monitoring of spreading depolarization pathology is an emerging strategy for personalized and targeted management of acute brain injury in neurointensive care. Spreading depolarizations (SDs) are pathologic waves that propagate slowly and focally through the cerebral cortex and other gray matter^1^. They are provoked by primary injury, such as stroke or trauma, and also by mismatches of energy supply–demand in tissue surrounding the primary injury^2^. As such, SDs recur for days to weeks after a primary injury and are associated with so-called secondary injury. Experimental studies have shown that they are a required causal mechanism for development of acute lesions such as ischemic infarcts^3^. 

SDs can be monitored in patients who require neurological surgery by placing a strip of electrodes directly on the brain for electrocorticographic (ECoG) recordings during intensive care^4,5^. Such recordings have shown that SDs are a predominant pathological mechanism in human brain injury and suggest that SDs should be targeted for treatment to improve outcomes. In traumatic brain injury, for instance, SDs occur in some 60% of patients and are independently associated with poor prognosis^6,7^. In patients with aneurysmal subarachnoid hemorrhage or ischemic stroke, the incidence is even higher at 80–100%^8,9^. The recently completed DISCHARGE-1 study has now shown that SDs are a significant mediator in the development of delayed infarction, independent of vasospasm, following aneurysm rupture^10,11^. On the basis of such studies, there are now two interventional trials underway to determine the feasibility and efficacy of SD monitoring to guide patient management^12^, and several neurosurgical centers are already using this monitoring in standard care management of patients. Ketamine is one line of defense to block SDs and thereby protect against lesion maturation or new infarct development^13–15^. This pairing of a real-time continuous diagnostic measure of neural dysfunction with an effective intravenous drug treatment offers hope for the realization of a “precision medicine” approach to neuroprotection, i.e., with adjustments of treatment dosing and duration that are tailored to the time course and prevalence of a specific mechanism in a specific patient^5,16,17^. The recent validation of less invasive techniques for ECoG monitoring may make this approach even more widely applicable^18,19^.

Nonetheless, a major barrier to adoption of SD monitoring remains: the difficulty of diagnosing these events in complex, multichannel ECoG recordings^20,21^. As a new field of clinical research and practice, the study and monitoring of SD requires very specialized training and experience that are outside the curriculum even for clinical neurophysiologists. In contrast to standard clinical practice, SD recordings are made with direct-current (DC)-coupled EEG amplifiers that record fluctuations down to the theoretical limit of zero Hz (DC)^20,22^. Such amplifiers are available now only after being developed specifically for SD research^17,23^. With recording of these slow and ultraslow potentials, there are vastly different timescales, amplitudes, and signal processing principles that must be understood before signals can be properly interpreted. Further, this requires mastery of new software as well as practical experience gained from exposure to large datasets. The lack of formal training opportunities and the scarcity of expert readers are major barriers to more widespread adoption of SD monitoring.

One approach to overcome these barriers is to develop automated methods to detect SDs in ECoG recordings. A software program, for instance, that could recognize SDs and provide real-time or summary reports for use in patient management could in principle supplant the need for specially trained ECoG readers. While EEG waveforms are notoriously difficult to decipher, even for the expert, SD traces have several prominent, unique features that might be easily recognized by a computational algorithm. These include (1) a negative shift of DC potential that is 10X the amplitude of seizures^24^ and lies in the unique frequency band of < 0.010 Hz, with average duration of ~ 2 min^25,26^, (2) a pronounced depression of spontaneous high-frequency ECoG activity (> 0.5 Hz) that develops simultaneously with the DC shift and typically persists for 8 min or more (aka, spreading depression)^26^, and (3) occurrence of these features at multiple electrode locations but (4) with a characteristic time delay that evidences the unique and identifying slow spread of SD through cerebral cortex. Jewell et al.^27^ have shown the promise of an automated approach to SD detection with the development of a software routine for bedside use.

Here we took a novel approach to automated SD detection by using the least amount of data required for reliable detection. In practice, this meant using only slow potential data from individual recording channels. Using training and test data sets that include over 3000 unique SD waveforms from brain-injured patients, this approach provides insight into the unique distinguishing features of these waves. Our results, showing high sensitivity and specificity in SD detection, further establish a robust and interpretable foundation for development of more complex, automated, and computational summary measures for clinical and research use.

## Materials and methods

### Data sources and ethics

Data consisted of ECoG recordings obtained from 24 patients enrolled in previous IRB-approved human subjects protocols. Surrogate informed consent or informed consent for study participation was obtained for all subjects. All data were de-identified prior to use in the present study, which was reviewed by the University of Cincinnati (UC) Institutional Review Board (protocol #2019–0548) and determined to be exempt. Cohort 1 consisted of 14 patients monitored at UC as part of a single-institution pilot study at UC under UC IRB #2013–4205 or as part of a multicenter study^6^ (ClinicalTrials.gov identifier: NCT00803036) under UC IRB #08–06-12–01. Cohort 2 consisted of 10 patients monitored at UC as part of a multi-center study under UC IRB #2016–8153. Cohort 1 was used to train and develop the machine learning model, while Cohort 2 was used as a naïve data set to test performance of SD detection methods. All research was conducted in accordance with the Declaration of Helsinki and results are reported in accordance with STROBE guidelines (https://www.strobe-statement.org).

### Electrocorticography acquisition and scoring

Methods for automated detection of SD were developed and validated using ECoG recordings obtained from brain injured patients according to established methods^5,7^. In brief, patients were admitted to neurosurgical centers for emergency treatment of severe brain trauma and, due to injury severity, underwent surgical treatment involving craniotomy. At the conclusion of surgery, a linear ECoG strip (platinum, 6-contact, 10-mm electrode spacing [Wyler; Ad-Tech]) was placed on the brain surface for subsequent recording of electrical activity (i.e., electrocorticography). Patients were transferred to the intensive care unit after surgery and continuous ECoG recordings were initiated. Recordings were acquired using a full-band amplifier (Advanced ICU Amplifier; Moberg ICU Solutions, Ambler, PA) at 256 Hz sampling rate and the Component Neuromonitoring System (Moberg ICU Solutions).

**Fig. 1:**
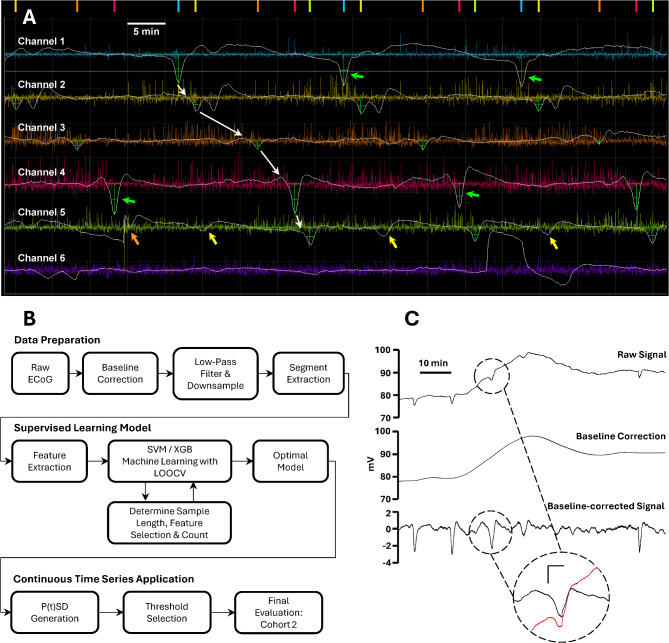
Methods of spreading depolarization scoring and model development. (**A**) Screenshot of OpenSD software shows the overlay of slow potentials (white) on high-frequency ECoG (colored traces; 0.5–50 Hz) for six channels of monopolar recordings. For slow potentials, OpenSD corrects the the direct-current (DC) baseline and drift by subtraction of the 30-min moving median voltage. (This differs slightly from the 10-min moving median used for baseline correction in the machine learning algorithm.) Ninety minutes of data are shown during which 4 spreading depolarizations (SDs) propagate from channel 1 to 5 (white arrows). The negative DC shift of each SD waveform is manually scored by use of cursor and keystroke, and waveforms are annotated by crosshairs. All annotations in the file are recorded and displayed above by raster marks corresponding to the channel color. All DC shifts of SDs are marked, including those more stereotyped (e.g. channels 1 and 4; green arrows) and those more atypical. Other waveforms that could be associated with SD, but lack definitive evidence (yellow arrows), or those corrupted by artifact (orange arrow) were not marked. (**B**) Flowchart of the major steps in data preparation, development of an optimal classification model through supervised learning, application to continuous time series data, and final evaluation. (**C**) illustrates the baseline correction of raw signals by subtraction of the 10-min moving median of the signal. The raw signal in the top trace shows a typical DC offset potential of > 70 mV with significant further drift. The offset and drift complicate data review and the drift can alter the waveforms of superimposed SDs, as shown in the inset (dashed circle, red trace). The middle trace shows the 10-min moving median which is subtracted from the raw trace for baseline correction. The resulting corrected signal (bottom trace) is centered at 0 mV with minimal, if any, distortion of the raw DC waveforms. Rather, the distortion introduced by DC drift is removed, making the waveform more easily recognizable by human and machine observers (see inset). Inset scale bars are 2 min and 2 mV.

For scoring, recordings were exported in European Data File format and then converted to Hierarchical Data Format version 5 for import into OpenSD software. OpenSD is a custom program developed in Python by the authors for the display, review, and scoring of SDs in full-band ECoG recordings. Each recording channel is displayed and processed in monopolar fashion vs. the external reference^23^. For each channel, slow potentials are overlayed on the band-pass filtered high-frequency (0.5–50 Hz) data for review of DC shifts together with depressions of spontaneous activity (Fig. 1A). To eliminate the DC offset and drift in the slow potential display, raw data are baseline-corrected by subtracting the median of the raw signal over a 30-min window bracketing (± 15 min) each data point. Moving median filters are nonlinear, time-domain filters that do not have closed-form analytic expressions to precisely define their frequency response; rather, the response is data-dependent. However, the chosen method is generally effective to attenuate fluctuations below the frequency band (0.001–0.010 Hz) of the typical DC shift of SD^26^. Because it preserves waveforms with minimal distortion, it is superior as a baseline correction method to the use of conventional filters with cutoffs of 0.005 or 0.010 Hz^20^. Moving median filters also provide a more smooth and effective correction of DC offset than, for instance, subtraction of a mean value over a given review time window (e.g. 1 h). The median is used, as opposed to the mean, to minimize the influence of brief, high-amplitude artifacts inherent to these recordings.

SDs were scored based on manual visual review of ECoG recordings in OpenSD software by an expert scorer (JAH). In conventional scoring^5^, each unique SD wave is counted based on review of all six electrodes of the strip. A single timestamp is assigned to each SD, which may include several waveforms occurring on different channels. By this conventional practice, multi-channel comparisons are necessary to identify the spread of an SD wave and are particularly informative when signals on some channels are weak, atypical, or partially corrupted. Here we used the same multi-channel comparisons and the established consensus procedures to identify SD waves. However, in contrast to standard procedure, here we annotated every individual SD waveform that occurred in every channel, marking the timestamp of the peak negativity (Fig. 1A). Thus, a single SD wave across the electrode strip could yield up to six individual waveforms. All waveforms were included, whether typical or atypical, and regardless of amplitude, provided there was certainty on its generation by SD. Waveforms were not scored if they were questionable or corrupted by artifact, or if, despite baseline correction, the negative peak of the DC shift remained above a zero-voltage value. Deciding on each individual waveform and electrode in this manner has never been performed previously for a cohort of patients. As such, there were no established or validated consensus criteria and scoring was inherently conservative with a known bias of excluding possible SDs.

## Data preparation

Raw ECoG data were prepared for use in machine learning and automated SD detection through several pre-processing steps, as follows. (1) *Baseline Correction*: Raw data were corrected for DC offset and drift by calculating the 10-min moving median of the raw signal. (Fig. 1C). The moving median signal was then subtracted from the raw signal. This 10-min method is slightly more aggressive than the 30-min moving median filter used for manual scoring in OpenSD software. (2) *Filtering*: Signals were then filtered using a finite impulse response (FIR) low-pass filter with a cutoff frequency at 0.5 Hz. (3) *Downsampling*: finally, data were downsampled from 256 to 1 Hz.

After preprocessing, segments of ECoG were extracted for use in supervised machine learning. Positive SD samples consisted of 1000-s segments centered on the timestamp of individual SD waveforms identified in expert manual scoring. Negative samples were derived from recording channels that had at least one SD and consisted of all non-overlapping 1000-s segments in which no SDs were present. With these criteria, 1548 positive (SD) and 13,416 negative (non-SD) samples were defined from patient cohort 1. Our preliminary work using machine learning to identify SD segments suggested that optimal performance obtained at 1 Hz down-sampling was maintained even when down-sampling segments to 0.1 Hz. Therefore, after extraction of the 1000-s segments, each segment was further down-sampled from 1.0 to 0.1 Hz, and all feature extraction in this study was performed on segments with this lower 0.1 sampling rate.

### Feature extraction

Feature extraction is the process of deriving computational measures (features) of the pre-processed, 0.1-Hz ECoG samples. These features were used in machine learning as input measures that may capture essential characteristics of SD waveforms and thus distinguish them from SD-negative samples. We took a comprehensive approach in exploring candidate features, using time- and frequency-domain measures that resulted in a feature vector of 80 dimensions or more for each ECoG sample. Time–frequency analysis methods, such as Discrete Wavelet Transform, were intentionally excluded from the feature set since (1) preliminary results suggested that time-domain and frequency-domain methods were adequate and (2) time–frequency methods would increase complexity and reduce computational efficiency while (3) providing little additional insight at 0.1 Hz sampling.

Candidate time domain features were (1) mean (μ = ΣX/N), (2) variance (σ^2^ = Σ(X—μ)^2^/N), (3) skewness (γ = E[(X—μ)^3^]/σ^3^), (4) kurtosis (K = E[(X—μ)^4^]/σ^4^ – 3), (5) entropy (H = -Σ[p(x)log(p(x)] for a signal with a probability distribution p(x)), (6) mean cross rate, a count of the times a signal crosses its mean value, (7) zero cross rate, a count of the times a signal crosses zero value, and (8–10) the three quartile values of a signal’s voltage distribution. These ten features were computed for the entire segment length and for sub-segments. Sub-segments were analyzed to explore features within more focused domains of longer signals. The sub-segments were defined as 30-sample lengths (300 s at 0.1-Hz sampling) iterated through the entire segment length with 10-sample overlap between consecutive sub-segments. Thus, for a segment length of 40 samples (400 s at 0.1 Hz sampling), 10 features were computed for the full segment and for two sub-segments, yielding a total of 30 features.

Both Fourier Transform (FT) and Power Spectral Density (PSD) were used to compute frequency-domain features. The FT is defined as F(ω) = ∫ f(t)e^(-jωt)^ dt and the PSD is calculated as the square magnitude of the FT. Features were defined based on the x- and y-coordinates of the five most prominent peaks in the FT and PSD, yielding 20 features. The absolute maxima of power/magnitude yielded two more. In addition, the axes of the FT and PSD were transformed into a Cartesian (coordinate) plane with rescaling of the axes to ranges of –1 to 1, and the number of peaks in each of the four quadrants for the FT and PSD yielded eight additional features. Finally, the ten time-domain features were computed for both FT and PSD, resulting in a total of 50 features.

### Supervised machine learning

We evaluated two prominent machine learning algorithms, Support Vector Machines (SVM) and Extreme Grading Boosting (XGB), to determine which was most effective in correctly classifying ECoG samples based on the defined features. Both algorithms have proven effective in the field of EEG signal analysis, notably in epilepsy diagnosis and classification^28,29^. The SVM operates by creating an N-dimensional hyperplane in a high-dimensional space that optimally distinguishes different classes of data points^30^. The key principle of kernel-based SVM is to map the data into a suitable higher dimension feature space in which the classes are linearly separable, and to maximize the *margin*, defined as the distance between the resulting separating hyperplane and the support vectors, the training samples closest to this hyperplane. The Radial Basis Function kernel, a popular choice for SVM due to its capacity to handle nonlinear aspects of the ECoG signals, was used in this study. In general, the maximum margin SVM approach guarantees best generalization. By contrast, XGB is a gradient boosting algorithm that constructs an ensemble of weak decision-tree learners, thereby often resulting in a model with superior predictive performance^31^. It manages missing values and outliers effectively, which is important in ECoG data where artifacts are common.

To evaluate and optimize machine learning models, we used the Leave-One-Patient-Out Cross-Validation (LOOCV) method. The model is trained using data from all subjects except one and model performance is tested on the remaining subject. The process is repeated using each subject as the test case and results are accumulated across all subjects. To evaluate candidate features, we used the XGB classifier to compute feature importance scores that quantify the relative contribution of each feature to the model’s predictive decisions. This score is based on average gain, defined as the improvement in the objective function at each tree split where the feature is employed, and reflects how strongly each feature influences the model’s internal partitioning of the feature space. The gain is calculated for each iteration of LOOCV, and the importance score is taken as the average gain across all iterations.

### Automated detection in continuous time series data

After development in supervised machine learning, algorithms for SD detection were applied to continuous ECoG time series data that have no pre-determined segmentation. For this purpose, ECoG data were pre-processed using identical steps as described above, including baseline correction, filtering, and down-sampling to 1 Hz. We then used the algorithm to compute probabilities of SD, *P*_*SD*_, for time windows of fixed length. For each window, data were further down-sampled to 0.1 Hz, features were computed, and a *P*_*SD*_ value was generated. The window was advanced iteratively in 1-s (or 10-s) increments and *P*_*SD*_ calculations were repeated to generate a time series, *P*_*SD*_*(t)*, with 1-Hz (or 0.1 Hz) resolution. Finally, the *P*_*SD*_*(t)* time series was evaluated for events that exceeded a threshold amplitude (*θ*_*P*_) for a threshold duration (*θ*_*D*_); these were considered SD detections. Optimal threshold values were determined empirically as described in Results.

## Performance evaluation in continuous time series

To evaluate performance on continuous ECoG data, automated SD detections were compared to expert manual scoring, which served as ground truth. For this purpose, a 400-s window centered on a ground-truth event was considered “positive SD”. If values in *P*_*SD*_*(t)* exceeded the amplitude threshold (*θ*_*P*_) for a threshold duration (*θ*_*D*_) within a 400-s “positive SD” window, they were considered true positive (TP) detections. Any *P*_*SD*_*(t)* events that exceeded threshold criteria outside these windows were considered false positives (FP). Any “positive SD” windows without aligned detection events were considered false negatives (FN).

Based on these counts, standard measures of sensitivity, specificity, accuracy, and precision were calculated. To evaluate the model, we used a novel objective function (OBJ) and the F1 score, defined as $F1 = \frac{{2 \times {\text{precision}} \times recall}}{{{\text{precision}} + recall}},$ which is particularly useful in the case of imbalanced data. OBJ, defined as $OBJ = TP^{2} - 2FP^{2},$ was used as a unique measure to evaluate performance based on both maximizing TPs and minimizing FPs. OBJ is biased against FPs by assigning them twice the penalty. This is important in scenarios such as SD detection where false alarms could lead to unnecessary medical intervention. Following the dictum, “first, do no harm,” we sought to bias the detection algorithm toward conservatism.

## Results

Our approach to developing an automated routine for SD detection is outlined in Fig. 1B. Raw ECoG time series data were prepared for computational analysis through steps of baseline correction, low-pass filtering, and down-sampling. We first used a supervised learning approach to develop a model that would optimally classify ECoG segments as SD-positive or -negative. To train the model, we used time-limited samples of single channel data centered on SDs identified in expert manual scoring of each of 6 ECoG channels in Cohort 1. This dataset consisted of 1128 h total recording time and included 1548 unique SD waveforms present in 11 of 14 patients. These served as positive training samples, while the remainder of data served as negative samples. After optimization, the model was adapted for application to continuous time series data. Further criteria for SD detection were specified and sensitivity/specificity were optimized. The final step was evaluation and validation on Cohort 2 data, consisting of 1187 h of recording and 1949 unique SD waveforms in 8 of 10 patients.

### Supervised machine learning

**Fig. 2:**
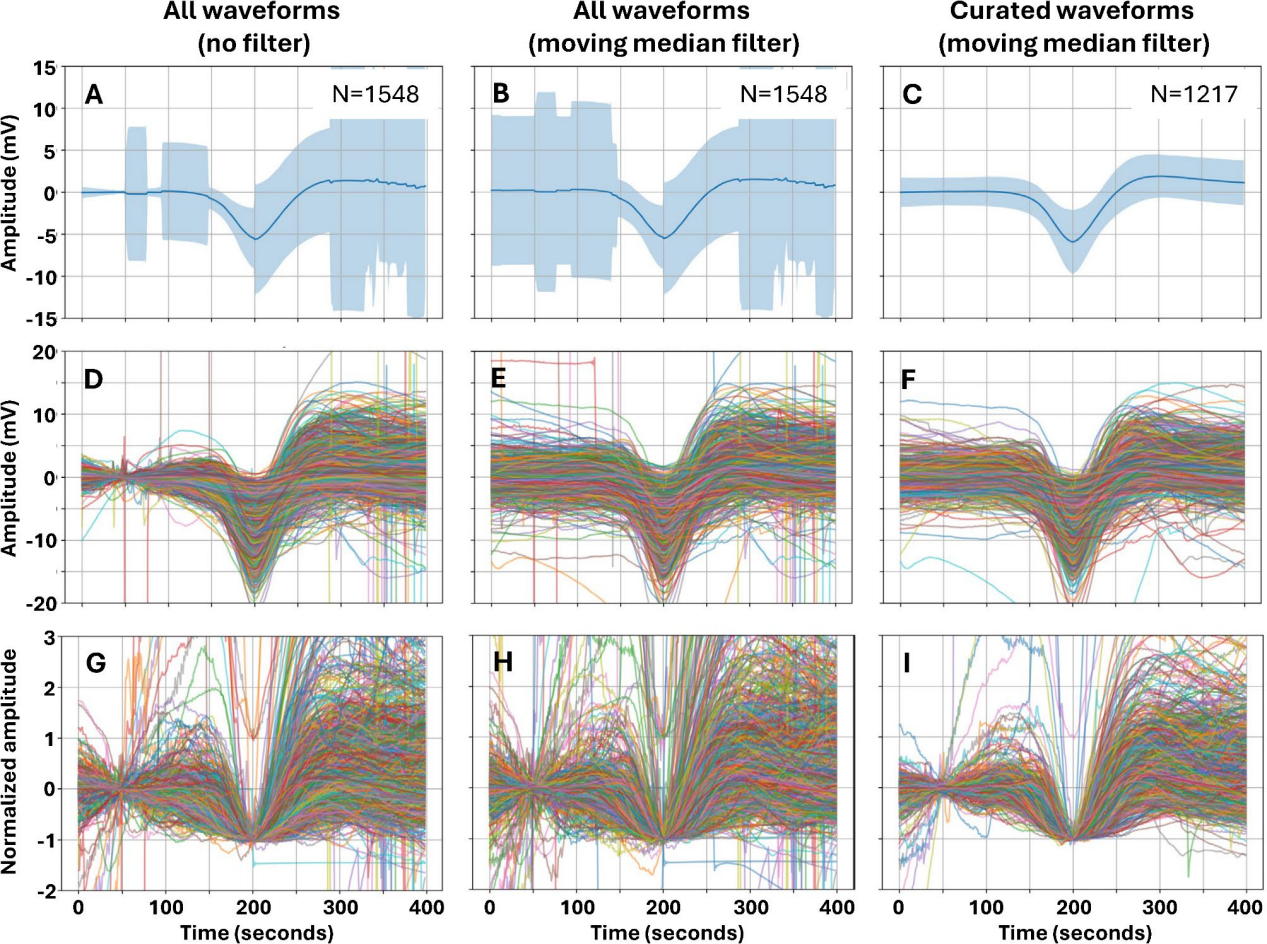
Waveforms of spreading depolarizations. Column 1 (**A, D, G**) shows all SD waveforms (n = 1548) identified in manual scoring of the Cohort 1 dataset. To correct DC offset, the median value over 0 to 100 s was subtracted. For comparison, **Column 2** (**B, E, H**) shows the same data but corrects for DC offset and drift by subtraction of the 10-min moving median. The peak negative amplitude of SD is 5.42 (± 3.55) mV without the moving median filter and 5.36 (± 3.77) mV with the median filter. The similarity shows that a 10-min moving median filter can correct DC baseline offset and drift without distorting or attenuating the average SD waveform. **Column 3** (**C, F, I**) is the same as column 2, but with removal of outlier waveforms to produce a curated dataset (n = 1217). All waveform samples were time-aligned at the peak of the negative DC shift of SD. **Row 1** (**A-C**) shows the mean and standard deviation of signals and **Row 2** (**D-F**) shows the individual waveforms. **Row 3** (**G-I**) shows waveforms after (i) subtracting the median value (0 to 100 s) to correct DC offset and (ii) normalizing amplitudes by setting the baseline voltage to zero and the peak voltage of the negative DC shift to -1.

The SD waveforms from Cohort 1 data (n = 1548) that were used as true-positive samples in supervised machine learning are shown in Fig. 2**.** Some samples included large DC shift artifacts that had the potential to corrupt and degrade training of a classification model. The dataset was therefore curated by removal of these outliers, resulting in 1217 samples. The average peak DC shift negativity in the curated dataset was -5.89 (± 3.79) mV. Normalization of negative DC shift amplitudes shows that waveform shapes conformed to a general stereotype but with considerable variation.

**Fig. 3:**
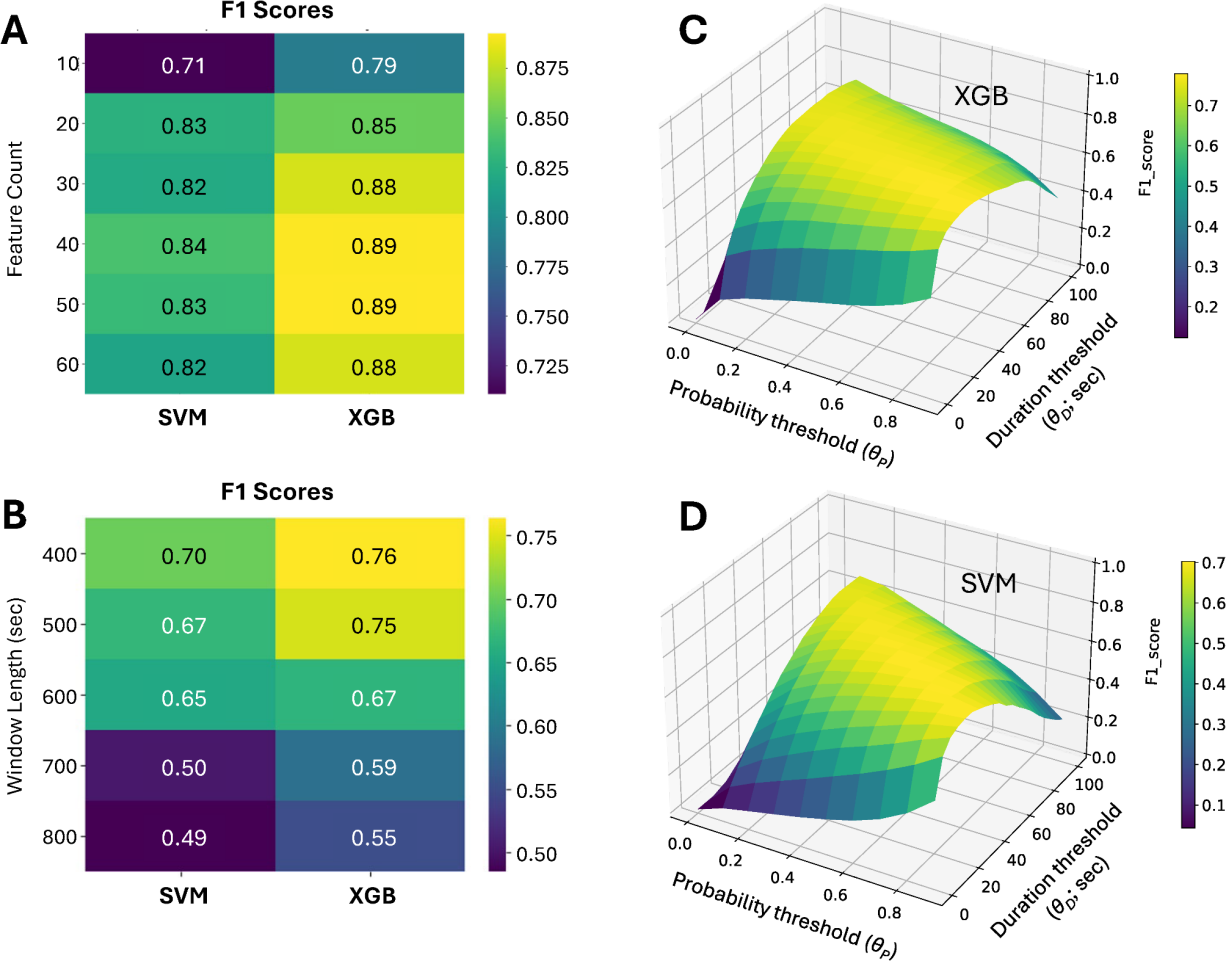
Selection of variables for optimal model performance. Heatmaps in (**A,B**) provide visual representations of F1 performance scores obtained using different feature counts (**A**) and segment lengths (**B**) in supervised machine learning with SVM and XGB models. Higher scores reflect better performance in terms of precision and recall balance. Different feature counts were tested using a segment length of 400 s and segment lengths were tested using a feature count of 30. 3D surface plots in (**C,D**) show algorithm performance (F1 scores) in continuous time series data for different combinations of probability threshold (*θ*_*P*_) and duration threshold (*θ*_*D*_).

We evaluated two prominent machine learning algorithms, XGB and SVM, for their ability to accurately classify ECoG training segments as SD-positive or -negative. The first step was to calculate candidate features for each segment (see Methods) as input variables for the classifiers and determine which features yielded optimal performance. Using the XGB classifier, we ranked and selected the top k features in order of importance in accurately classifying 400-s segments. We then trained models using different feature counts (k) and evaluated performance using LOOCV. Results showed that performance, based on F1 scores, was nearly optimal using 20–30 features, with only marginal further gains by including 40–50 features (Fig. 3A). We next experimented with different segment lengths to determine whether optimal performance could be maintained, or even enhanced, with shorter segments, since the typical DC shift of SD is only ~ 2 min (Fig. 2). Using LOOCV, we found that performance improved at shorter lengths and was best at 400 s (Fig. 3B). Segment lengths less than 400 s were not investigated since 40 data points (0.1 Hz sampling of 400-s segments) were considered a minimum for feature computation. Finally, we found only negligible differences in performance when training models on the full vs. curated datasets.

In these experiments, we found that XGB consistently outperformed the SVM algorithm. Thus, the final model selected was the XGB method using segment lengths of 400 s and 30 features. The lower feature count was chosen in order to limit computational load and, by minimizing the risk of overfitting, to optimize the model’s generalization ability. The final step in model development was hyperparameter optimization, which was accomplished by a grid search of values and performance evaluation by LOOCV. The final model had accuracy of 0.98, precision of 0.91, recall of 0.89, and AUC of 0.99 in classifying SD-positive and -negative samples.

### Distinguishing features of spreading depolarizations

The selection of features for use in the machine learning algorithm can be considered a first step toward an objective, quantitative characterization of the SD waveform. Of the 30 features used in the final model, 29 had significantly different distributions between the SD-positive and -negative samples used in the training set (*P*’s < 0.002; Mann–Whitney with Bonferroni correction). Eleven features were in the time domain and mainly included measures of the distribution of voltage values such as kurtosis, skewness, standard deviation, and quartile values. Cross rates for zero and mean voltages were also included. The remaining features were in the frequency domain and related either to measures of the frequency-amplitude distribution (kurtosis, skewness, standard deviation, and quartiles) or to X and Y coordinates of the peaks of the distribution. These quantitative features all correspond intuitively to the visual features used in expert manual ECoG scoring to identify SDs. By design, they relate mainly to the large negative shift of DC potential.

**Fig. 4:**
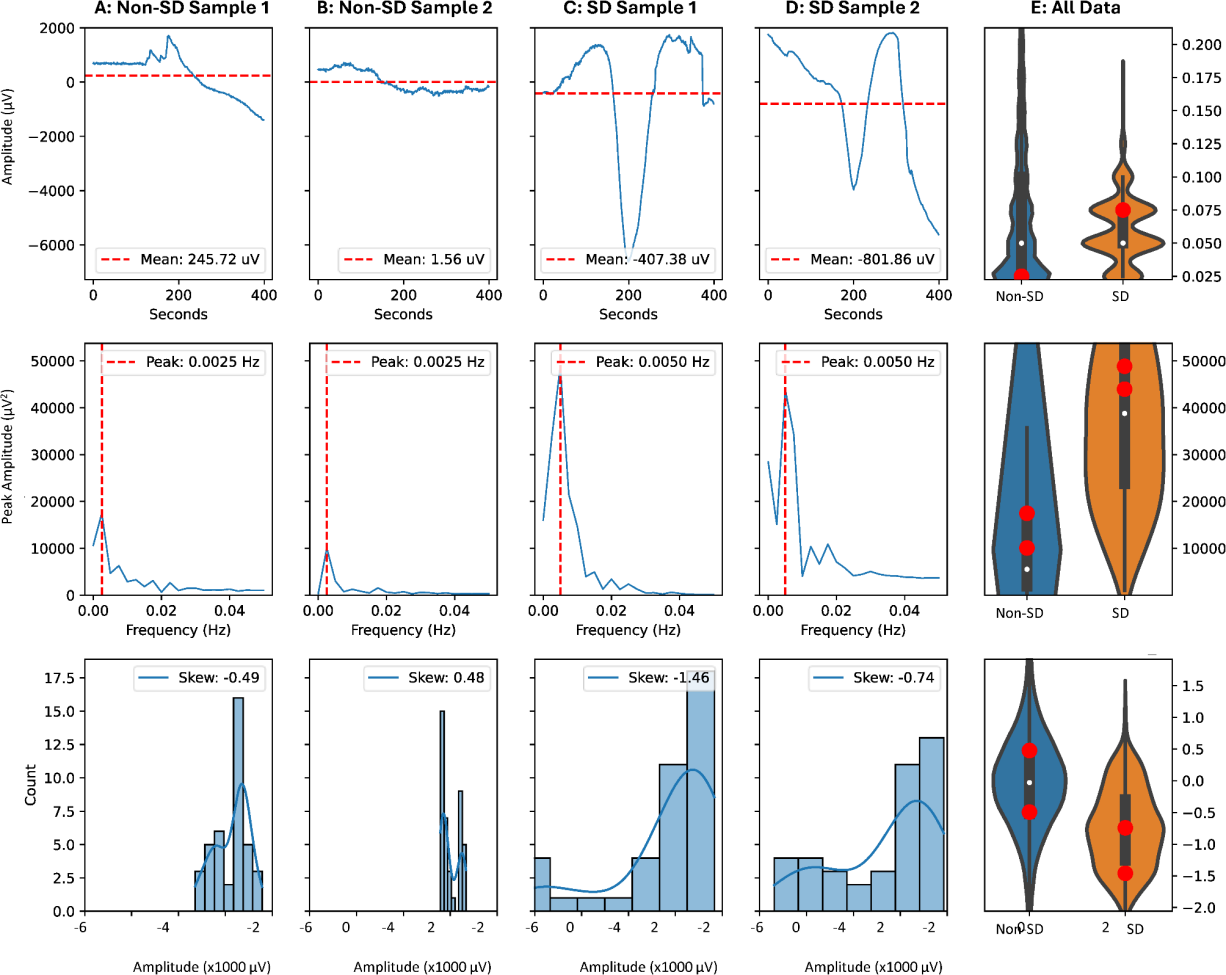
Distinguishing features of spreading depolarizations. The first four columns show representative features for two non-SD (**A,B**) and two SD (**C,D**) electrocorticography segments. The first row shows the raw waveforms after subtracting the 10-min moving median and illustrates the higher mean cross rate for SDs. The second row shows the fourier transforms of the signals, illustrating the higher peak amplitudes for SDs. The third row shows the distributions of amplitude values, illustrating the greater skewness for SDs. The violin plots (**E**) show smoothed kernel density estimates of the distributions of all data in the training sets. The median (white symbol) and interquartile range (thick line) are shown and the thin lines (whiskers) extend beyond the quartiles by 1.5 times the range from median to quartile. The red symbols show the values for the two samples illustrated for non-SD and SD events. The median values (interquartile range) for the mean cross rate were 2 (2, 3) for SD samples and 2 (1, 4) for non-SD. While the medians were identical, the different shapes of the distributions contributed to a high importance score for this feature in discriminating positive and negative samples. Median values for peak amplitude of the fourier transform were 38,784 (23,727, 54,175) for SD and 6089 (2379, 15,746) for non-SD, and for skewness -0.79 (-1.26, -0.29) and -0.05 (-0.56, 0.45).

Figure 4 presents SD-positive and -negative examples to illustrate the correspondence of quantitative and visual features. The large negative DC shift of SD occurs at a median peak frequency of 2 mHz (IQR 2–5), which is the same peak frequency found in the baseline DC signal when no SDs are present (median: 2; IQR 2–2). Despite the overlap in frequency bands, SDs are distinguished by a greater peak amplitude (**middle row**). This large negativity results in greater negative skewness of the distribution of amplitude values when SD occurs, as compared with more narrow and normal distributions around zero when there is no SD (**bottom row**). It also typically results in a greater number of crossings of the mean value of a sample segment (**top row**) since the negative shift and return to baseline define at least two crossings, and positivities before and after can result in more. When no SD is present, single mean-crossings are the most common.

For these and all other features, there was considerable overlap in distributions for SD-positive and -negative ECoG segments, as illustrated in the violin plots of Fig. 4. This overlap is attributable to the diversity of SD waveforms, and particularly the inclusion of less stereotypical “edge” cases that may, for instance, have smaller amplitudes. It is also due to the presence of DC noise and the sensitivity of ECoG electrodes to biological variables that induce signals with similarities to SD. Thus, SD-positive and -negative samples are not easily and reliably distinguished by individual features or even small numbers of features; larger features sets are required.

### Application to continuous time series data

**Fig. 5:**
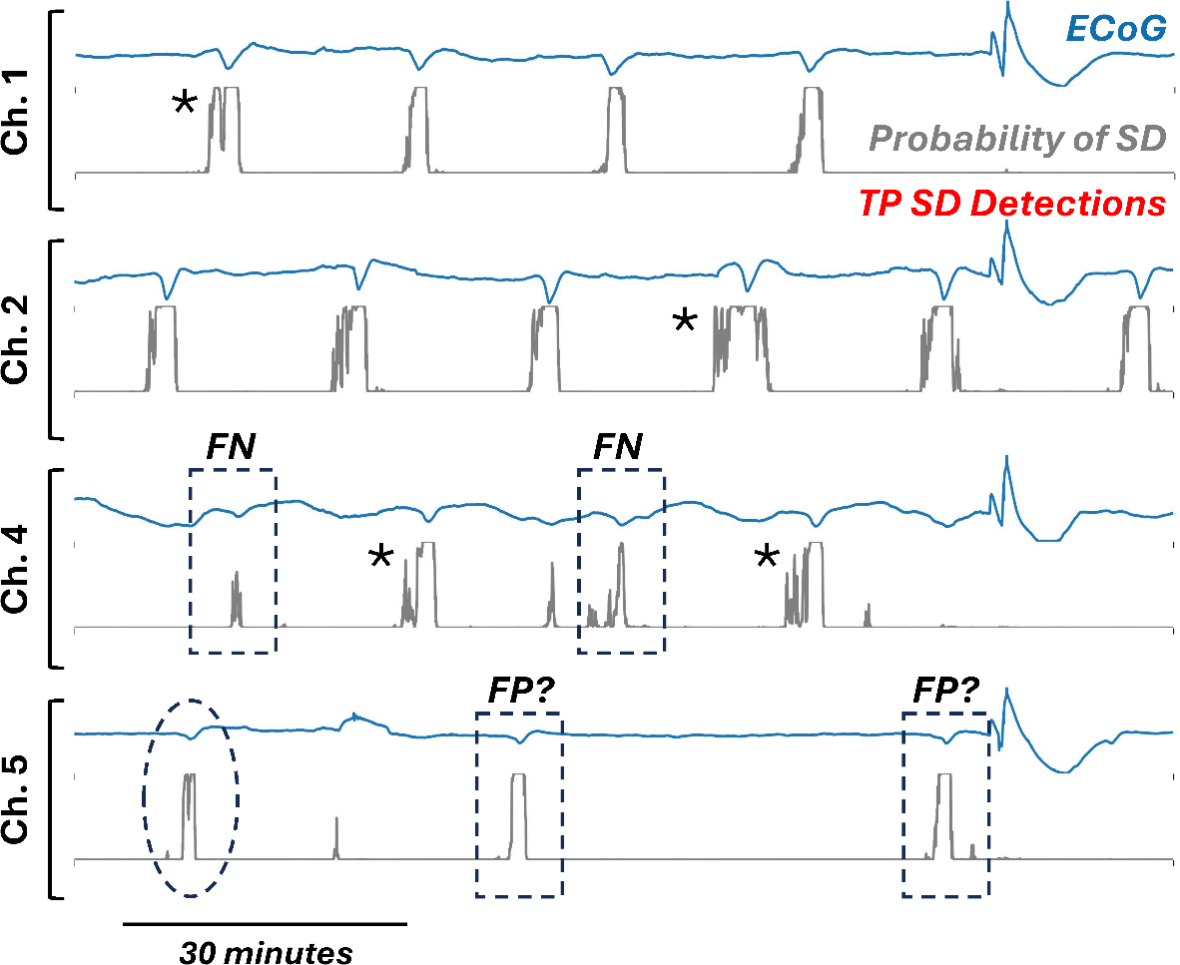
Adaptation of detection algorithm to continuous time series. The model developed in supervised machine learning was used to generate a times series (gray) for probability of SD, *P*_*SD*_*(t)*, based on sliding window assessment of the pre-processed ECoG data (blue). When *P*_*SD*_ exceeds a threshold value (*θ*_*P*_) continuously for a threshold duration (*θ*_*D*_), an SD is detected (red bars). Representative data are shown for 2 h of ECoG recording in 4 of 6 monopolar, pre-processed channels. Four SDs in Channel 1 and six in Channel 2 are easily recognizable in manual scoring; all were accurately detected by the algorithm and there were no false positives. In Channel 4, two SDs were accurately detected by the algorithm but two were missed and scored as false negatives (FN; dashed boxes). Both FNs triggered elevated *P*_*SD*_ values but did not reach thresholds for SD detection. In Channel 5, with much smaller ECoG signals, two SDs were detected but were counted as false positives (FP; dashed boxes) since they were not annotated as SDs in expert manual scoring. However, post-hoc adjudication confirmed that these were SDs. A third event in Channel 5 (dashed oval) was missed by both algorithm and expert scorer. Asterisks denote SDs that triggered multiple peaks.

The model developed using supervised machine learning makes binary decisions (SD or not) and was trained on positive examples from a curated dataset with the DC shifts of SDs centered in the limited data segments. Thus, several steps were necessary to adapt this classification algorithm to operate on continuous ECoG recordings that have no pre-determined segmentation. Our approach was to use the algorithm to calculate the probabilities of SD for windows of data with fixed length of 400-s that moved progressively through the continuous data in 1-s increments, thus generating a probability time series, *P*_*SD*_*(t)* (Fig. 5). Initial experience in examining *P*_*SD*_*(t)* traces revealed a large number of spurious high probability values that were false positives. The high number of these events was attributable to the sheer volume of segments being evaluated in consecutive 1-s increments (e.g. 518,400 in 1 day of 6-channel data) as compared to the small number of negative samples (13,416) used in supervised learning. Furthermore, individual SDs resulted in high *P*_*SD*_ values that were elevated over tens of 400-s windows (seconds) and sometimes caused multiple *P*_*SD*_ peaks (see asterisks in Fig. 5). These difficulties precluded use of any simple read-out of SD detections based on *P*_*SD*_ threshold crossings.

Therefore, it was necessary to set thresholds for both the probability value (*θ*_*P*_) and for the duration (*θ*_*D*_) for which probability was above *θ*_*P*_. Any event in the *P*_*SD*_ time series exceeding both thresholds would be considered an SD. To determine optimal values for *θ*_*P*_* and θ*_*D*_, all 1128 h of continuous recording data from Cohort 1 were processed to generate corresponding *P*_*SD*_ time series. The *P*_*SD*_ data were then evaluated to generate SD predictions using a range of *θ*_*P*_ and *θ*_*D*_ values. The performance of these threshold combinations was evaluated against the ground truth of expert manual scoring by using the F1 score. Figure 3C,D show the results for the XGB and SVM methods using 30 features and 400-s window lengths. Both graphs show optimal performance at a range of *θ*_*P*_ and *θ*_*D*_ combinations, extending from short durations at high probabilities to long durations at low probabilities. Raw counts of true positives, false negatives, and false positives for representative threshold combinations suggest a generally high level of performance in SD detection (Table 1). For instance, the algorithm detects 70–80% of the 1548 SDs identified in ground-truth manual scoring, and false positive rates range 5–13 events/day across all six ECoG channels combined. As in supervised learning, performance of the XGB model was superior.

**Table 1:** Model performance on training and test cohorts.

	**Model**		**Duration threshold (*θ***_***D***_**)**	**Probability threshold (*θ***_***P***_**)**	**F1 score**	**OBJ** **function** *(*× *10*^*5*^*)*	**True positives** *count (%)*	**False negatives** *count (%)*	**False positives** *count (#/day)*	**Adj. false positives** *count (#/day)*
**Cohort 1**	**XGB**	Cumulative	40	0.7	0.773	11.6	1132 (73)	416 (27)	244 (5.1)	
			20	0.8	0.769	11.5	1252 (81)	296 (19)	426 (9.0)	
			50	0.5	0.764	11.0	1100 (71)	448 (29)	222 (4.7)	
			60	0.3	0.759	10.7	1086 (70)	462 (30)	218 (4.6)	
	**SVM**	Cumulative	40	0.7	0.706	8.1	1064 (69)	484 (31)	381 (8.0)	
			50	0.5	0.702	7.4	1093 (71)	455 (29)	461 (9.7)	
			60	0.3	0.694	5.8	1145 (74)	403 (26)	589 (12.4)	
			20	0.8	0.689	4.6	1164 (75)	384 (25)	633 (13.4)	
**Cohort 2**	**XGB**	Cumulative	50	0.5	0.709	13.6	1252 (64)	697 (36)	323 (6.5)	99 (2.0)
		Subject 1					148 (60)	97 (40)	104 (14.4)	27 (3.7)
		Subject 2					0 (0)	2 (100)	30 (6.5)	27 (5.9)
		Subject 3					352 (72)	140 (28)	46 (3.9)	9 (2.2)
		Subject 4					0 (N/A)	0 (N/A)	4 (0.5)	4 (0.5)
		Subject 5					259 (83)	55 (17)	27 (14.8)	2 (1.1)
		Subject 6					11 (26)	31 (74)	13 (1.6)	8 (1.0)
		Subject 7					222 (55)	178 (45)	28 (9.3)	10 (3.3)
		Subject 8					110 (51)	107 (49)	58 (16.1)	12 (3.3)
		Subject 9					150 (63)	87 (37)	13 (8.2)	0 (0.0)

Table shows accuracy of SD detection in both datasets. For Cohort 1, used in model development, both XGB and SVM models were tested using various combinations of probability and duration thresholds. Representative results are shown. Cohort 2 was used as a test dataset for the selected XGB model with *θ*_*P*_ = 0.5 and *θ*_*D*_ = 50 s. Models were tested on all data. (cumulative) in the corresponding cohorts and were scored against events identified in expert manual scoring as ground truth positives. True positives reflect the number (percentage) of these events that were detected by the algorithm; the remainder are false negatives. False positives are the algorithm-detected events that were not identified in expert manual scoring. For Cohort 2, results are shown for both the cumulative dataset and for individual subjects. For this test dataset, false positive counts were adjusted (**Adj.**) following post-hoc adjudication of events identified as false positives in the initial performance test. Upon manual re-review of the 323 false positives, 224 were evaluated to be possible, probable, or definitive SDs that were not scored as definitive SDs in initial manual scoring. The false positive count is thus reduced to 99. Duration thresholds are in seconds. Rates reported in events per day of 6-channel recordings.

### Evaluation in continuous time series data

Based on results above, the XGB model using 400-s sample length and 30 features was selected as the final model for performance testing and validation on a naïve data set, Cohort 2. For this purpose, we selected *θ*_*P*_ = 0.5 and *θ*_*D*_ = 50. While other threshold combinations had slightly better performance on F1 and OBJ scores, this combination had a low rate of false positives and this aspect was prioritized for conservative scoring. Table 1 shows that algorithm performance on the naïve dataset was sustained at a high level, with 64% of 1949 SDs identified as true positives. The false positive rate was 6.5 SDs/day. Importantly, the algorithm performance was relatively consistent across individual subjects. The percentage of true positives detected ranged 51 to 72 across 6 of 7 subjects that had considerable event counts; the algorithm was less sensitive in one case with only 26% of true positives detected. Although performance overall deteriorated somewhat in comparison to the training Cohort 1, this is to be expected based on some degree of over-fitting the model to data in the training cohort with corresponding loss of generalization.

**Fig. 6:**
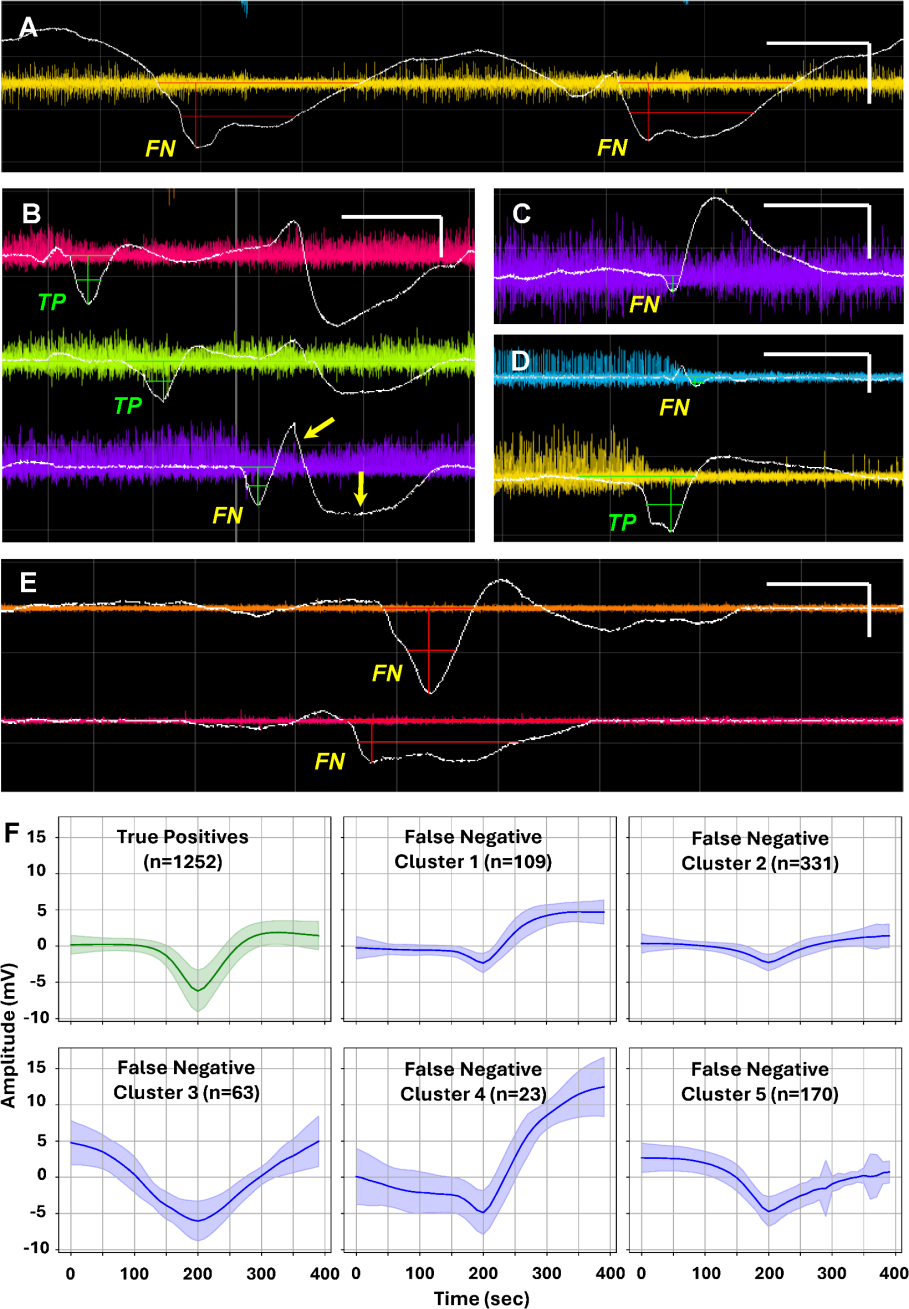
False negatives in test cohort. Panels **A-E** show representative examples of SD waveforms that were not identified by the algorithm, i.e. false negatives (FN). Those identified by the algorithm on adjacent recording channels are labeled as true positives (TP). For each channel, slow potentials (white) are shown after baseline correction by subtraction of the 30-min moving median voltage and are overlayed on high-frequency ECoG (colored traces; 0.5–50 Hz) of the corresponding monopolar channel. Scale bars show 5 min and 5 mV. (**F**) shows means and standard deviations for all true positives and false negatives in cohort 2 testing. False negatives were separated into five clusters using the* k*-means method based on the waveforms of pre-processed segments. These clusters illustrate trends of deviation from more stereotyped SD waveforms, including low amplitude negativities (clusters 1 and 2), high amplitude positive after-potentials (clusters 1 and 4), and prolonged durations (clusters 3 and 5).

To better understand the algorithm’s limitations, all events that were not detected by the algorithm, i.e. false negatives, were informally reviewed by visual inspection. In the majority of cases (426/697; 61%), reasons for lack of detection were not easily discernable. However, 240 cases (34%) had waveforms that were starkly atypical due to prolonged durations of the negative DC shift (Fig. 6A,E), low amplitude of the negative DC shift (Fig. 6C, D), large amplitudes of the positive afterpotential (Fig. 6C), or other anomolies. This is similar to results from a prior study in which an identical 34% of SD waveforms were characterized as atypical^23^. The often consistent nature of these variations is evident in plots of false negative waveforms that clustered together (Fig. 6F). Finally, in 31/697 (4%) cases there was artifact interference with the waveform, which may have adversely impacted detection (Fig. 6B).

**Fig. 7:**
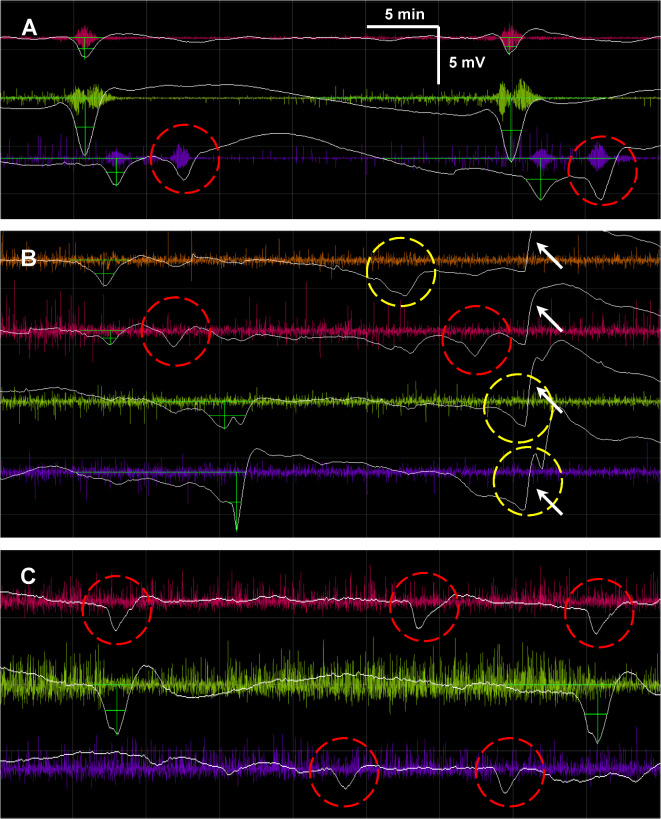
“False positives” in test cohort. Panels **A-C** show representative examples of waveforms (red dashed circles) that were identified by the algorithm as SDs but were not scored manually. For each channel, slow potentials (white) are shown after baseline correction by subtraction of the 30-min moving median voltage and are overlayed on high-frequency ECoG (colored traces; 0.5–50 Hz) of the corresponding monopolar channel. Green crosshairs indicate waveforms that were identified in manual scoring as SDs. In (**A**), the purple channel exhibits a rare “double-hump” waveform phenomenon that is sometimes encountered. While these are SDs based on (i) the propagation pattern across the strip, (ii) their stereotyped repetition, and (iii) the pulse artifact observed in sparsely active tissue (see also green and red channels), it is uncertain whether both or one of the humps should be considered the SD waveform. In (**B**)**,** the double-hump is encountered in another patient (red channel) and the algorithm identifies both humps as SDs, in contrast to the manual scorer. Another SD spreads across the electrode strip 20 min later but waveforms (yellow dashed circles) were not scored manually due to the presence of an artifact (white arrows) that distorts the waves on the green and purple channels. Nonetheless, the SD was identified on the red channel by the detection algorithm. In (**C**), several probable SDs were identified by the algorithm on the red and purple channels that did not reach the threshold for more conservative manual scoring, possibly due to lack of stereotypy in the propagation pattern or unconvincing ECoG depression. In all cases **A-C**, the algorithm-identified “false positives” represent likely or definitive SDs. Time and voltage scale bars apply to slow potential traces in all panels.

False positives in the test cohort were similarly adjudicated in a secondary manual review of recordings by an expert scorer. This was particularly justified by the conservative bias in the initial manual scoring of ground truth, which excluded possible or likely SDs that would then appear as false positives in automated scoring. This bias was intentional and even necessary, since it is acknowledged that aspects of how individual SD waveforms are presented in human ECoG are incompletely understood. Confirming this, review of the “false positives” detected by the algorithm showed that a majority of events (224/323; 69.3%) were definitely (n = 126), probably (n = 50), or possibly (n = 48) real SDs and not spurious false detections made by the algorithm. As shown in the examples of Fig. 7, these events did not conform to conservative criteria based on more typical SD waveforms and thus were not scored as SD in the initial manual scoring. However, they did have many hallmark characteristics of SDs and could be rightly considered SDs. When the algorithm’s performance is re-scored based on this post-hoc adjudication, the false positive rate drops to an impressive 2.0 events/day (Table 1). Importantly, low false positive rates were observed across all subjects.

Finally, while the continuous application was developed and tested using 1-Hz resolution for the *P*_*SD*_*(t)* time series, we also examined performance using the same sparse 0.1-Hz sampling as was used in feature computation. Results for Cohort 2 using the XGB model with *θ*_*P*_ = 0.5 and *θ*_*D*_ = 50 were nearly identical for both sampling frequencies. At 0.1-Hz, the OBJ function score was 13.1 × 10^5^ with 1,247 true positives, 702 false negatives, and 347 false positives (c.f. Table 1, Cohort 2, Cumulative for 1-Hz sampling). This sensitivity test demonstrates that performance is not dependent on use of 1-Hz data, but rather can be achieved with sparse data sampling and greater computational efficiency.

## Discussion

Manual scoring of SDs in ECoG recordings is complex. It requires training on specific software, some understanding of signal processing, and exposure to large datasets to gain experience in pattern recognition. Moreover, when trained, review of ECoG to identify these events – and distinguish them from imposters – requires the simultaneous assessment of multiple recording channels as well as multiple data displays across a broad spectrum of frequencies. By contrast, here we have shown that only sparse data sampling is required to identify SDs when taking a computational and algorithmic approach based on machine learning. Namely, we demonstrated that only a single voltage reading every 10 s (0.1 Hz) from a single channel is required to identify SD. When applied to a naïve dataset, this minimalist approach was able to identify 64% of true positives identified by an expert scorer with only two false positives per day (i.e., per 144 recording hours = 24 h × 6 channels). These results demonstrate that accurate bedside, automated detection of SDs is possible. They further suggest a maximum required temporal sampling frequency, which may have implications for non-invasive approaches to SD detection using optical imaging or other methods.

By precluding the need for expert bedside personnel to make SD diagnoses, automated software-based solutions would overcome a major barrier to more widespread adoption of ECoG monitoring. The approach developed here, for instance, has already been applied with practical benefit in SD scoring. The automated detection algorithm has been integrated into the novel OpenSD software that has been custom-built for the offline processing, display, and scoring of SDs in ECoG recordings. The software can thus be used to score SDs either manually or by the algorithm, and the results of algorithmic detection can be reviewed and edited manually for hybrid machine-human scoring. These routines and functionalities are also being incorporated into live-streaming software to allow for real-time automated SD detection that could be used for bedside decision-making in neurointensive care. Real-time implementation is eminently feasible, since computation time to execute the algorithm on one hour of six-channel pre-processed ECoG data using a standard laptop ranged from 84.6 to 3.2 s, depending on the sampling and stride rates used. The low end of this range was achieved using rates of 0.1 Hz, thus validating the computational advantage of our minimalist sampling approach. Importantly, automated detection may offer not only a savings of manual labor, but also possibly an improvement in scoring accuracy, since the detection algorithm in some respects performed comparably to expert manual scoring. In testing on the naïve cohort 2 dataset, the algorithm detected 323 events that were not identified as SDs in manual scoring and thus were considered false positives. However, secondary manual review of these events showed that 224 were definitely, probably, or possibly real SDs. This result confirms the conservative nature of manual scoring and suggests that automation may be a comparable, or at least complementary, approach.

Our approach to SD detection differed vastly from the typical and recommended approach of human intelligence in manual ECoG review. Manual visual identification of SD takes account of all ECoG information available, including (1) multiple channels of simultaneous recording to evidence spread, (2) both slow potentials (< 0.05 Hz) for recognition of DC shifts and higher frequency activity (0.5–50 Hz) for recognition of spreading depression, and (3) comparison of events through time to assess for stereotypy of waveforms on individual channels. In contrast to this approach, here we attempted to detect SDs using the most limited information possible, i.e. 40 data points of single-channel data with only 0.1-Hz sampling. Given the success in doing so under such severe constraints, this approach now allows the progressive construction of even more robust detection algorithms through the use and incorporation of additional ECoG information. This includes, for instance, the use of multi-channel information, the assessment of time delays between channels, and the algorithmic detection of spreading depression. In view of these future aims, the number of false negatives (36% of all ground-truth positive SDs) obtained with the algorithm is not concerning. Multi-channel ECoG and the spreading nature of depolarizations result in an inherent redundancy of information when the entire electrode strip is considered, such that a false negative in one channel would likely be compensated by true-positive detections in other channels for the same SD wave (e.g. Figure 6B,D). With this in mind, our approach was intentionally conservative, favoring avoidance of false positives at the expense of increased false negatives. Furthermore, apart from this intentional design, the number of false negatives was not surprising, considering that ground truth positive events were identified in expert scoring with the aid of multi-channel and full bandwidth information, as compared to the sparsely sampled, single channel data available to the algorithm. It is possible that the future incorporation of multi-channel information in detection decisions may increase the sensitivity and specificity of the algorithm to a performance level that may entirely preclude the need for manual review of software-based detections.

Our approach also differed from the standard practice, in both manual scoring and the automated approach of Jewell et al.,^27^ of identifying unique SD waves across the electrode strip. In these approaches, all channels are considered to determine if a new wave occurred, and this wave is annotated as a single SD “on the strip,” inclusive of each waveform on different channels and irrespective of the number or identity of channels in which the SD was manifested. The identification of “an SD” is thus a superordinate label that is applied to all recordings from an electrode array. In this customary practice, it is not necessary to decide definitively whether waveforms on individual electrodes are SDs or not. Here, by contrast, we endeavored to score each individual SD waveform on each electrode throughout all 24 patient recordings in our datasets. This exercise was worthwhile in highlighting the variety of slow potential SD waveforms and, particularly, in calling attention to the edge cases with very low amplitude or atypical morphology. These are usually ignored in customary scoring practice since adequate evidence of SD can be obtained from other channels with more robust, stereotyped waveforms. Inclusion of all SD waveforms in the training data set is likely to have contributed to greater accuracy and generalizability in algorithm performance.

The present study is the first to score and characterize the DC shifts of all SD waveforms. The unbiased, automated approach that we developed may avoid the pitfall of human intelligence that attends more to the best (most stereotyped) examples of SDs. Human intelligence is biased toward simplification, generalization, and rule formation, and therefore also underestimates heterogeneity. This can lead to an overly dogmatic and simplified understanding of the waveform characteristics of SDs. For example, while DC shifts typically have a prominent negative phase followed by a slight positivity (Fig. 2), exceptions and variations on this generalization are plentiful (Figs. 6,7). These variants include waveforms that have (i) very small amplitude, (ii) two negative phases, (iii) more prominent positive than negative phases, or (iv) very prolonged negativities or positivities. Similarly, while SDs typically induce spreading depression of spontaneous activity (0.5–50 Hz), objective study of SDs after aneurysmal subarachnoid hemorrhage showed that spreading depression occurs in only 65% of cases. The remaining SDs were associated with no change or even an increase in spontaneous activity^32^. Thus, caution is warranted against overly simplistic rules or characterizations of SDs, and more research is needed to understand the mechanisms and significance of the diversity of patterns in which SDs are manifested. The present study provides a first step toward such understanding by providing automated and objective methods to identify SDs. This can lead to the development of large databases and further quantitative study.

A limitation of the specific algorithm developed here is that it can only be applied to recordings obtained with full-band (“DC”) amplifiers – the same amplifiers used to acquire the data used in algorithm training. Before such amplifiers were available, it was common practice to monitor for SDs with AC-coupled amplifiers with a lower frequency limit in the range of 0.010 Hz. While these amplifiers are still used at several centers, they severely distort and attentuate the DC shifts of SD, which have a peak frequency (0.002 Hz) that is well below this lower limit. DC amplifiers, by contrast, record unfiltered waveforms, have high signal-to-noise ratios, and are generally recommended as the gold standard. Another possible limitation is that our methods were developed using data only from patients with traumatic brain injury. SDs are a stereotyped phenomenon that depend on local conditions of cerebral cortex, and not the triggering stimulus or disease. Therefore the consensus criteria^5^ for recognizing SDs in the human brain apply to a variety of conditions including ischemic stroke, spontaneous intracerebral hemorrhage, and aneurysmal subarachnoid hemorrhage. Given this stereotypy, we expect that the developed methods would generalize to use for ECoG monitoring of any patient, regardless of underlying disease or SD etiology. However, this remains to be validated. The addition of more patient cohorts to the dataset for algorithm training, including those with different brain injury types, should result in more robust algorithm performance and greater generalizability.

## Data Availability

The data that support the findings of this study may be available from the corresponding author upon reasonable request.
